# Impact of simulated gastric acid on electrochemical behavior, surface morphology, and topography of 3D printed cobalt chromium and titanium alloys for dental applications

**DOI:** 10.1186/s12903-026-08639-2

**Published:** 2026-05-25

**Authors:** Kawkb M. El-Tamimi, Dalia A. Bayoumi, Mohamed M. Z. Ahmed, Mohamed I. A. Habba, Sherif E. Zahra, Mohammed E. El-Sayed

**Affiliations:** 1 Department of Removable Prosthodontics, Faculty of Dentistry, Suez Canal University, Ismailia, 41522 Egypt; 2 Department of Dental Biomaterials, Faculty of Dentistry, Suez Canal University, Ismailia, 41522 Egypt; 3 Department of Metallurgical and Materials Engineering, Faculty of Petroleum and Mining Engineering, Suez University, Suez, 43512 Egypt; 4 Department of Metallurgical and Materials Engineering, Faculty of Engineering, Atatürk University, Erzurum, 25240 Turkey; 5 Mechanical Department, Faculty of Technology and Education, Suez University, Suez, 43518 Egypt; 6 Department of Orthodontics, Faculty of Dentistry, Suez Canal University, Ismailia, 41522 Egypt

**Keywords:** Salivary pH, 3D printing, Co-Cr and Ti-6Al-4V alloys, Corrosion morphology, Topography, Gastroesophageal reflux disease (GERD)

## Abstract

**Background:**

Additive manufacturing of cobalt–chromium (Co–Cr) and titanium (Ti-6Al-4 V) alloys is increasingly used in prosthetic dentistry. Acidic oral environments associated with gastroesophageal reflux disease (GERD) may negatively influence the surface integrity and corrosion resistance of dental materials. This in vitro study assessed the effects of simulated gastric acid on the corrosion resistance, surface morphology, and topographical changes of selective laser melting (SLM) Co–Cr and Ti-6Al-4 V alloys.

**Methods:**

Thirty-two additively manufactured specimens (*n* = 16 per alloy) were randomly distributed into acidic (pH 1.2) and neutral control (pH 6.7 distilled water) subgroups (*n* = 8). A cyclic static immersion protocol was used to simulate GERD-related acid exposure. Specimens in the acidic group were immersed for 2 min in acidic solution, rinsed without scrubbing to preserve the delicate surface topography, and stored in distilled water at 37 °C, repeated six times daily over nine days with a 24-hour interval maintained between each daily cycle. Control specimens were stored continuously in distilled water at 37 °C. Surface morphology, elemental composition, and topography were evaluated using scanning electron microscopy (SEM) and Abbott–Firestone curve analysis.

**Results:**

In the Co–Cr alloy, immersion in an acidic solution resulted in a decrease in the percentage of all elements, except O, which increased to 6.37 ± 1.77%, with the change being statistically significant (*P* ≤ 0.05). In contrast, Ti-6Al-4 V also showed a decrease in all elements after immersion, with O rising to 2.26 ± 1.22%, although no changes were statistically significant (*P* ≥ 0.05). SEM images indicated more oxide deposits on Co-Cr in an acidic solution, which also showed a notable increase in surface roughness, whereas Ti-6Al-4 V exhibited greater stability. The Abbott–Firestone analysis further confirmed that Co-Cr underwent more significant changes in peak formation and exploitation zones than Ti-6Al-4 V did in acidic environments.

**Conclusions:**

Under simulated gastric acid exposure, Ti-6Al-4 V alloys exhibited superior corrosion resistance and surface stability compared with Co–Cr alloys. These findings support the clinical preference for Ti-6Al-4 V in dental restorations for patients experiencing frequent acidic challenges, such as those with severe GERD.

## Introduction

Gastro-esophageal reflux disease (GERD) is characterized by symptoms and esophageal mucosal alterations resulting from the reflux of gastric contents into the esophagus [1]. In Western populations, GERD affects approximately 10–20% of individuals [2]. Gastroesophageal reflux, typically experienced as heartburn, occurs in 40%–85% of pregnant women [3]. GERD decreases oral fluid pH, creating an acidic environment that can adversely affect dental materials, particularly metals [2, 4, 5]. A salivary pH of 5.5 or lower is especially significant, as it promotes metal corrosion [6]. Cobalt-chromium and titanium alloys are commonly employed in dental restorations and orthodontic devices due to their long-term corrosion resistance, attributed to a protective passive oxide layer [7]. These alloys form a thin oxide barrier (approximately 1–10 nm), primarily consisting of Cr2O3 in cobalt-chromium and TiO2 in titanium alloys [8]. Cobalt-chromium alloys offer advantages such as low cost, high modulus of elasticity, high strength, low flexibility, high melting point, and low density [9]. Titanium and its alloys possess low density, low Young’s modulus, high mechanical strength, low thermal conductivity, low electrochemical corrosion rate, and are less expensive than traditional noble metals like gold [10]. Current challenges for metallic biomaterials include improving bio-functionality and reducing manufacturing costs [11]. CAD-CAM technology has eliminated traditional waxing, investing, and casting steps, streamlining the fabrication of complex structures [12]. Additive manufacturing (AM), notably selective laser melting (SLM), has rapidly advanced in this field [13]. SLM produces metal substrates with minimal porosity by melting alloy powder layer by layer [14]. Sahen et al. investigated the electrochemical corrosion behavior, ion release, and surface hardness of Co-Cr and Ti-6Al-4 V alloys fabricated using CAD-CAM milling and laser sintering. CAD-CAM milling demonstrated superior corrosion resistance compared to laser sintering. Additionally, the Co-Cr alloy exhibited lower corrosion and ion release than the Ti-6Al-4 V alloy [13]. Bechir et al. assessed the corrosion behavior of two commercial Co-Cr dental alloys produced by casting and milling in artificial saliva at pH levels of 3, 5.7, and 7.6. The milled Co-Cr alloy showed enhanced corrosion resistance, supporting its preference for prosthetic management in patients with GERD [15].

Previous studies have evaluated corrosion behavior mainly through electrochemical testing alone or by combining electrochemical measurements with basic SEM imaging, without extending the analysis to quantitative surface topography characterization. Such approaches, while revealing, provide an insufficient picture of alloy degradation, as electrochemical parameters reflect bulk corrosion kinetics but do not capture the spatial distribution of surface damage, oxide deposition patterns, or load-bearing topographical changes that are directly relevant to the clinical performance of dental restorations when exposed to simulated gastric acid (pH 1.2) in GRED patients. Therefore, the present study was conducted with the following objectives: (1) evaluate and compare the electrochemical corrosion behavior of 3D-printed Co-Cr and Ti-6Al-4 V alloys in simulated gastric acid (pH 1.2) and neutral (pH 6.7) solutions; (2) evaluate the changes in elemental composition and oxide formation on the alloy surfaces following acid exposure using energy-dispersive X-ray spectroscopy (EDX); (3) analyze and compare the surface morphology and roughness of both alloys under acidic and neutral conditions using scanning electron microscopy (SEM) and surface roughness profiling; (4) analyze the surface topographical changes of both alloys using Abbott-Firestone analysis, with particular focus on peak formation, exploitation zones, and void distribution; and (5) determine which alloy demonstrates superior corrosion resistance and surface stability under simulated GERD conditions, thereby informing evidence-based material selection for dental applications in patients with acidic oral environments. Null Hypothesis (H₀): There is no significant difference in corrosion resistance, surface roughness, elemental composition, or surface topography between 3D printed Co–Cr and Ti-6Al-4 V alloys when exposed to simulated gastric acid (pH 1.2) compared to neutral conditions.

## Materials and methods

### Samples preparation

Cylindrical specimens with dimensions of Ø12 × 5 mm were printed using selective laser melting process. Titanium specimens were fabricated utilizing titanium grade 5 Ti-6Al-4 V powder (TC4, BLT) and a 3D printer (A160 BLT). Particle size from 15 to 45 μm, powder layer thickness of 25 μm, fiber laser power of 200 W, focus diameter of approximately 75 μm, argon working atmosphere, no pre-heating, hatch distance of 0.105 mm, checkerboard hatch pattern, scan speed of 1250 mm/s, contour offset of 0.0825 mm, and power supply of 1.5 kW. Following the SLM processes, the specimens were annealed at 820 °C (10 °C/min) for 4 h in a furnace under an argon atmosphere and then gradually cooled to room temperature. For the CoCr samples, the specimens were processed with 10 to 30 μm alloy powders type 5 (Starbond Easy Powder Scheftner) using a 3D printer (Vulcantech VM120). Laser spot diameter of 0.08–0.1 mm, a scanning speed of 1100–1200 mm/s, and a layer thickness of 0.02 mm. Table 1 shows the brand names, manufacturers, and elemental compositions of Co-Cr and Ti-6Al-4 V alloys. The specimens underwent heat treatment in a furnace using high-purity argon after the SLM procedures. At a ramp rate of 10 °C per minute, the specimens were heated from room temperature to 1150 °C and maintained there for six hours in the furnace, then gradually cooled to room temperature, and subsequently polished with felt wheels to achieve an enhanced surface finish.

**Table 1:** Brand names, manufacturers, and elemental compositions of Co-Cr and Ti-6Al-4 V alloys

Co-Cr Alloy		Ti-6Al-4 V Alloy	
Starbond simple Pulver 30, Scheftner dentistry, Germany		TC4 BLT, China	
El.	Wt. %	El.	Wt.%
Co	61	Ti	90
Cr	27.5	Al	5.5–6.75
W	8.5	V	3.5–4.5
Si	1.6	Fe	> 0.3
C, Fe, Mn	> 1%	C	> 0.08
		N	> 0.05
		H	> 0.015
		O	0.08 − 0.015

### Experimental design

The computation of the sample size was adopted from an earlier study on the corrosion of Ti-6Al-4 V and Co–Cr alloys [13] and performed using G Power version 3.1.9.2, Faulet al [16]. Based on an effect size of f = 0.94, with α = 0.05 and β = 0.05 (power = 95%), the required total sample size was 24 samples across four groups. In this study, 8 samples were included in each group (total *n* = 32), which exceeds the minimum required sample size and thereby provides additional statistical robustness. The samples were divided into two main groups (*n* = 16 each) of Co-Cr and Ti-6Al-4 V alloys. Each main group was further subdivided into two subgroups (*n* = 8 each) based on the immersion solution used: simulated gastric acid (pH 1.2, subgroup B SG-B) and a control (distilled water, pH 6.7, subgroup A SG-A). This 2 × 2 factorial design allowed both intra-alloy (acidic vs. neutral conditions) and inter-alloy (Co-Cr vs. Ti-6Al-4 V) comparisons across different pH environments.

### Treatment protocols

Simulated gastric acid solution (pH 1.2) was prepared according to the British Pharmacopoeia by dissolving 2.0 g NaCl and 7.0 mL of concentrated HCl in 1 L of distilled water. The experimental methodology was implemented based on previous studies with modifications to mimic the intermittent nature of acid exposure in the oral environment. Specimens for each alloy were randomly assigned into two subgroups (*n* = 8) according to the pH of the immersion medium: an acidic group (pH 1.2) and a neutral control group (pH 6.7 distilled water). To replicate the erosive challenges observed clinically in Gastroesophageal Reflux Disease (GERD), a cyclic static immersion model was employed. For the acidic subgroup, samples underwent a controlled cycle: they were immersed in the acidic solution for two minutes, rinsed with distilled water without scrubbing to preserve the delicate surface topography, and subsequently stored in distilled water at 37 °C. This procedure was repeated six times daily over a period of nine days, with a 24-hour interval maintained between each daily cycle. The control group was maintained in a continuous static state in distilled water at 37 °C.

Before each immersion cycle, a calibrated digital pH meter with an accuracy of ± 0.01 was used to measure the pH of the distilled water. It was always between 6.5 and 6.8, which is the natural pH of distilled water in equilibrium with atmospheric CO₂. The pH was recorded throughout the experimental period to ensure stability and uniformity of the control condition. No chemical adjustment of the distilled water pH was performed, as this range corresponds to a biologically neutral condition and is corresponding with the control solutions employed in similar corrosion studies of dental alloys.

### Electrochemical experiments

Electrochemical experiments were conducted using a GAMRY Reference 3000 potentiostat/galvanostat/ZRA analyzer. A classical three-electrode setup was employed, which consisted of the sample under test as the working electrode (exposed surface area of 0.25 cm²), a saturated calomel electrode (SCE) as the reference electrode, and a Pt grid as the counter electrode. The electrochemical cell was filled with either a simulated gastric acid solution (pH 1.2) or distilled water (pH 6.7) as the electrolyte. A thermostatic water bath maintained the temperature at 37 ± 1 °C. Before testing, the samples were cleaned with ethanol, rinsed with distilled water, and dried with compressed air. Each specimen was immersed in the test electrolyte, and the open-circuit potential (OCP) was monitored for 1 h before any electrochemical measurement. OCP data were acquired at a sampling interval of one data point per second using the GAMRY Framework™ software. The OCP drift rate had to be less than 1 mV per minute over the last 10 min of the stabilization period for a steady-state condition to be reached. This requirement verified that a stable electrode–electrolyte interface had been established before the following electrochemical measurements. Electrochemical impedance spectroscopy (EIS) measurements were performed at the stabilized OCP by applying an AC sinusoidal excitation signal of 10 mV amplitude (peak-to-zero) over a frequency range of 100 kHz to 10 mHz, with 10 data points collected per decade. The resulting Nyquist and Bode plots were analyzed and fitted using the GAMRY Echem Analyst™ software to extract equivalent circuit parameters, including charge-transfer resistance (Rct) and double-layer capacitance (Cdl). Following EIS measurements, potentiodynamic polarization tests were performed to determine the corrosion current density (i_corr_) and corrosion rate (CR). The potential was scanned from − 250 mV to + 250 mV relative to the stabilized OCP at a scan rate of 1 mV/s. Using Tafel extrapolation, the i_corr_ values were determined by fitting the linear parts of both the anodic and cathodic Tafel slopes (βa and βc). The CR was calculated from the i_corr_ values using Faraday’s law, accounting for the equivalent weight and density of each alloy. For each tested material, a single electrochemical measurement was carried out to determine the corrosion parameters.

### Surface morphology and topography analysis

A scanning electron microscope (SEM) (Prisma E, Thermo Fisher Scientific, Waltham, MA, USA) equipped with an integrated energy-dispersive X-ray spectroscopy (EDX) unit was used to examine the surface morphology and elemental composition of the specimens. SEM imaging was performed at an accelerating voltage of 30 kV and a beam current of approximately 1.0 nA. Elemental composition analysis was performed using an integrated silicon drift detector (SDD) EDX unit (Thermo Fisher Scientific) under the same accelerating voltage of 30 kV. EDX spectra were collected over an energy range of 0–20 keV, with a live acquisition time of 60 s per spectrum and a spectral resolution of approximately 127 eV at Mn Kα. Surface topography was evaluated using the Abbott–Firestone technique, also known as the material ratio curve or bearing ratio curve, which provides a cumulative probability distribution of surface heights as a function of material ratio, offering quantitative insights into the load-bearing capacity, void distribution, and peak characteristics of the alloy surfaces. Following the immersion protocols, the surface profiles of all tested specimens were collected using a calibrated contact profilometer under standardized measurement conditions. The raw surface profile data were transferred to Microsoft Excel for further computation and graphical representation of the Abbott–Firestone curves. The curves were divided into three functional zones: the peak zone, the core exploitation zone, and the void zone. The percentage contribution of each zone to the total surface profile was calculated and recorded for all specimens under both acidic (pH 1.2) and neutral (pH 6.7) conditions. The total roughness parameter (Rt), which is the vertical distance from the deepest valley to the highest peak in the measured profile, was also determined from the Abbott–Firestone curves.

### Statistical analysis

An independent t-test was used for intra- and intergroup comparisons between the two types of solutions (neutral and acidic) and between the two types of alloys (Co-Cr and Ti-6Al-4 V). Differences were considered statistically significant (95% significance level). A p-value ≤ 0.001 was considered to be highly statistically significant (99% significance level). The test was selected because the comparisons were limited to predefined pairwise groups. The Shapiro–Wilk test was used to test the normality of the data. Data were analyzed using statistical software SPSS version 25 (IBM Co., USA).

## Results

### Electrochemical behavior

**Fig. 1:**
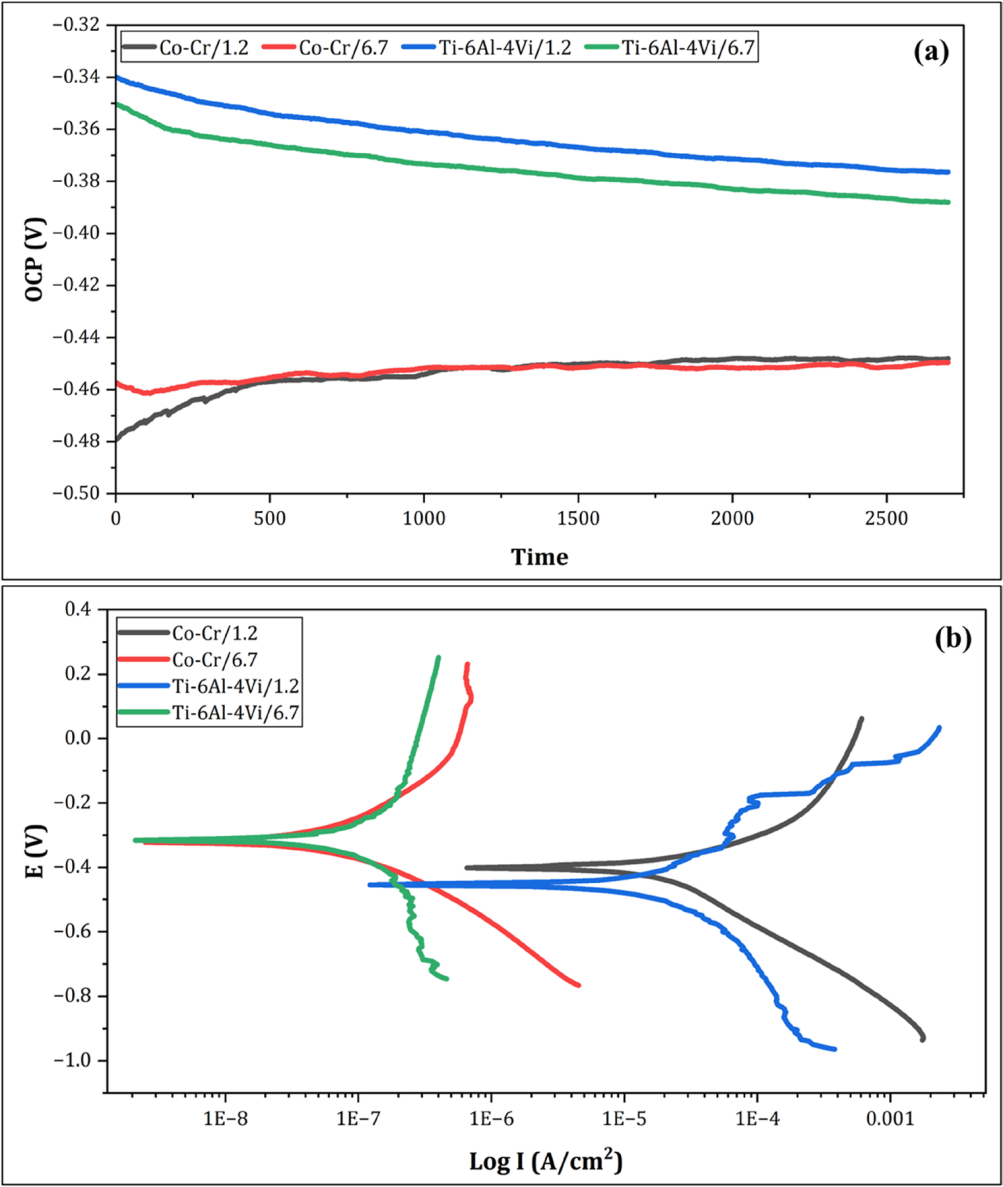
(**a**) OCP vs. time plots and (**b**) potentiodynamic polarization curves for both Co–Cr and Ti-6Al-4 V alloys in acidic (pH 1.2) and neutral (pH 6.7) solutions

Figure 1a shows the OCP evolution of Co–Cr and Ti-6Al-4 V alloys recorded over 3600 s of immersion in both acidic and neutral solutions before going to electrochemical testing. In the acidic solution, both alloys showed an initial transient period characterized by a gradual shift in OCP, followed by progressive stabilization toward steady-state values. The Co–Cr alloy in the acidic solution stabilized at a more negative OCP of approximately − 0.46 V, whereas Ti-6Al-4 V in the same medium achieved a less negative steady-state potential of approximately − 0.38 V. In the neutral solution, both alloys showed a more positive and stable OCP from the outset, with Co–Cr stabilizing at approximately − 0.47 V and Ti-6Al-4 V reaching approximately − 0.37 V. The OCP drift rate satisfied the criterion of less than 1 mV/min over the final 10 min of stabilization for all tested conditions, confirming that a stable electrode–electrolyte interface was determined before subsequent measurements. The less negative OCP values recorded for Ti-6Al-4 V under both pH conditions suggest a more thermodynamically stable surface than Co–Cr. Figure 1b shows the potentiodynamic polarization curves of the Co–Cr and Ti-6Al-4 V alloys in both acidic and neutral solutions, plotted as potential versus logarithmic current density. In the acidic solution, the Co–Cr alloy exhibited a higher anodic current density and more active corrosion behavior, with the polarization curve displaying a less defined passive region. In contrast, Ti-6Al-4 V in the acidic solution showed a more extended and stable passive plateau, reflecting the superior resistance of its TiO₂ passive film to electrochemical dissolution in acidic conditions. In the neutral solution, both alloys exhibited reduced current densities across the entire potential range, indicating enhanced passivation. However, Ti-6Al-4 V maintained a more noble corrosion potential and broader and more stable passive region. The Tafel-derived corrosion parameters extracted from these curves are listed in Table 2. Collectively, the polarization behavior of both alloys confirmed that Ti-6Al-4 V exhibited superior corrosion resistance under both tested pH conditions, with the difference being most pronounced in the acidic environment simulating gastric acid exposure.

**Fig. 2:**
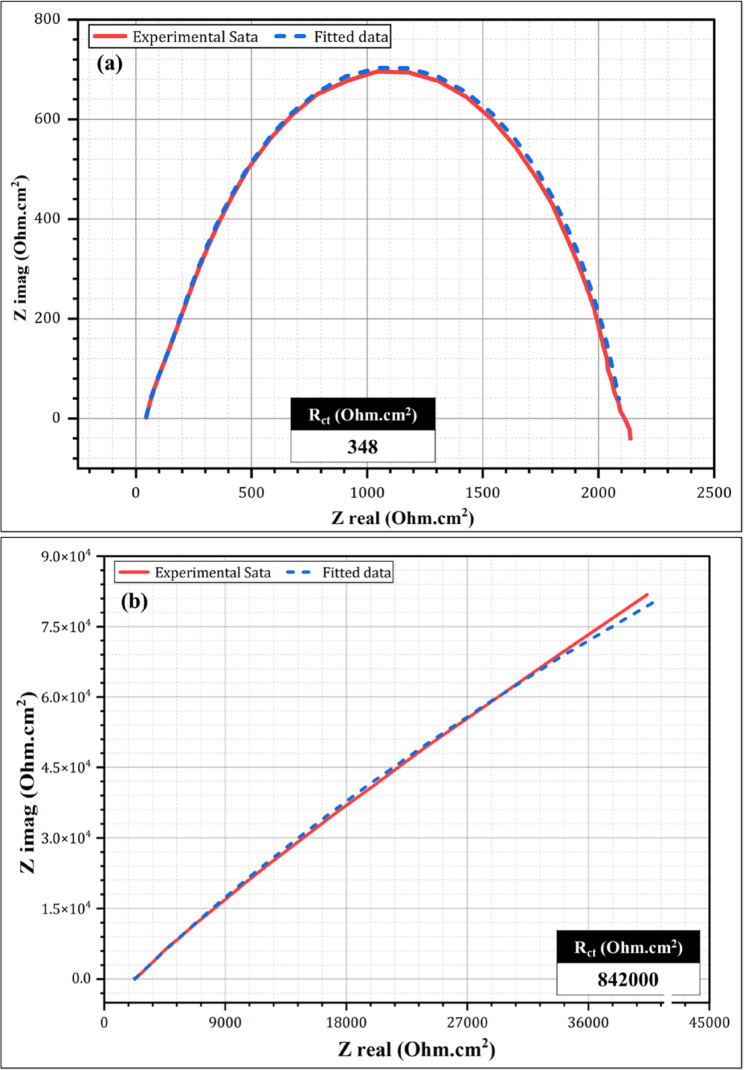
Nyquist plots (experimental and fitted data) of the Co–Cr alloy after immersion tests in (**a**) acidic and (**b**) neutral solutions

**Fig. 3:**
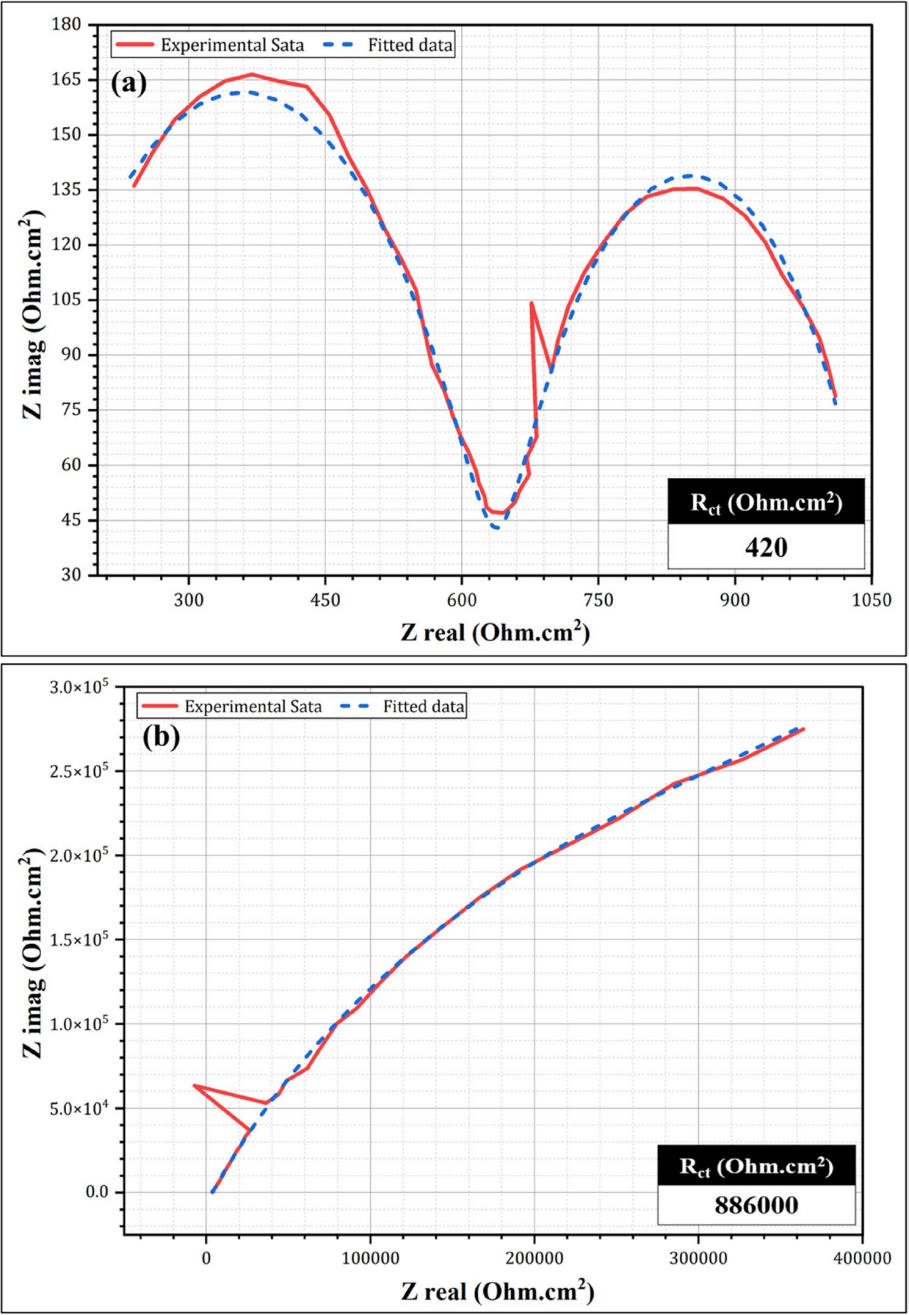
Nyquist plots (experimental and fitted data) of Ti-6Al-4 V alloy after immersion in (**a**) acidic and (**b**) neutral solutions

Table 2 presents the electrochemical behaviors of the Co-Cr and Ti-6Al-4 V alloys in acidic and neutral solutions. The Ti-6Al-4 V alloy demonstrated superior corrosion resistance compared to the Co-Cr alloy in acidic and neutral environments. In the acidic solution, Ti-6Al-4 V exhibited a lower corrosion current density (i_corr_) of 20.7 µA/cm^2^ compared to 37.2 µA/cm^2^ for Co-Cr (Table 2), indicating better corrosion resistance than Co-Cr. This trend was even more pronounced in the neutral solution, with Ti-6Al-4 V exhibiting an i_corr_ of 0.122 µA/cm^2^ compared to 1.6 µA/cm^2^ for Co-Cr. The corrosion rate (CR) values were significantly lower for Ti-6Al-4 V in both solutions, especially in the neutral solution, where it showed a CR of 0.158 mpy compared to 1.4 mpy for Co-Cr (Table 2). The results listed in Table 2 indicate that the Ti-6Al-4 V alloy is more resistant to corrosion and surface alterations in acidic and neutral solutions. Figures 2 and 3 show the electrochemical impedance spectroscopy (EIS) results for the Co-Cr and Ti-6Al-4 V alloys, respectively, in both acidic (pH 1.2) and neutral (pH 6.7) solutions. It can be remarked that for the Co-Cr (Fig. 2) and Ti-6Al-4 V alloys (Fig. 3), there is a stark contrast between their behavior in acidic and neutral solutions. In the acidic solution (Fig. 2a), the Nyquist plot of Co-Cr shows a small, depressed semicircle with a diameter of approximately 350 Ω. cm². This indicates a relatively low charge transfer resistance, suggesting that the Co-Cr alloy is more susceptible to corrosion in acidic solutions. The depressed nature of the semicircle can be attributed to oxide formation under acidic conditions. In contrast, the Co-Cr alloy in the neutral solution (Fig. 2b) exhibited a much larger semicircle with a diameter exceeding 800 kOhm.cm². This significant increase in the diameter of the semicircle indicates a substantially higher charge transfer resistance, implying an enhanced corrosion resistance in a neutral solution. The near-perfect semicircular shape suggests the formation of a more uniform and stable passive layer on the alloy surface under neutral conditions. Figure 3 illustrates the EIS results for the Ti-6Al-4 V alloy. In the acidic solution (Fig. 3a), the Nyquist plot revealed a semicircle with a diameter of approximately 420 Ohm.cm². Although this is larger than that of Co-Cr under acidic conditions, it still indicates some vulnerability to corrosion. The Ti-6Al-4 V alloy in the neutral solution (Fig. 3b) demonstrated greater corrosion resistance, with a semicircle diameter of approximately 886,000 Ohm.cm². This value was slightly higher than that of Co-Cr under neutral conditions, indicating superior corrosion resistance. The near-perfect semicircle shape implies a highly stable and uniform passive layer on the Ti-6Al-4 V surface in a neutral solution. Comparing the two alloys, Ti-6Al-4 V consistently showed better corrosion resistance than Co-Cr under both acidic and neutral conditions. This is evidenced by the larger semicircle diameters of both solutions. The difference was particularly pronounced in the acidic solution, where Ti-6Al-4 V exhibited a significantly higher charge transfer resistance.

**Table 2:** Corrosion results of Co-Cr and Ti-6Al-4 V alloys after immersion test in acidic and neutral solutions

Solution	Sample	i_corr_ (µA/ cm^2^)	CR (mpy)
1.2	Ti-6Al-4 V	20.70	18.34
	Co-Cr	37.20	34.04
6.7	Ti-6Al-4 V	0.122	0.158
	Co-Cr	1.60	1.40

Table 3 shows the EDX results for the Co-Cr and Ti-6Al-4 V alloys after immersion testing in acidic and neutral solutions. For the Co-Cr alloy, a statistically significant increase in oxygen content was recorded from 3.83% in the neutral solution to 6.37% in the acidic solution (*p* < 0.05), indicating a measurable change in the surface oxygen-containing species under acidic conditions. While this finding is consistent with the electrochemical evidence of increased corrosion activity, the EDX data are reported as semi-quantitative surface measurements and are interpreted accordingly, shown in Table 2 and Figs. 1 and 2. In contrast, the Ti-6Al-4 V alloy showed a smaller, non-significant increase in oxygen content from 0.69% to 2.26% when exposed to an acidic solution (*p* > 0.05). This relatively minor change in oxygen content corresponds to the superior corrosion resistance of the alloy, as detected in the electrochemical behavior of Ti-6Al-4 V, as presented in Table 2. The stability of the Ti-6Al-4 V surface composition, particularly the limited increase in oxygen, contributed to its better performance in acidic solutions. The intergroup comparison of the oxygen content between the two alloys was statistically significant in both neutral (*p* = 0.002) and acidic (*p* = 0.008) solutions. This significant difference in oxide formation correlates with the distinct corrosion behavior observed in the electrochemical tests. Therefore, previous results indicate that while both alloys experience some degree of oxidation in acidic solutions, the Ti-6Al-4 V alloy maintains better surface stability and corrosion resistance. This is reflected in both its electrochemical behavior and the limited changes in its elemental composition, particularly the oxygen content, when exposed to simulated gastric acid.

**Table 3:** Mean ± SD of the percentage of elements (wt%) in different solution pH values for both alloys

Alloy	Element	Neutral	Acidic	*P*-value*
**Co-Cr**	C	6.23 ± 1.81	6.21 ± 2.28	0.990^NS^
	O	3.83 ± 2.24	6.37 ± 1.77	0.036^S^
	Cr	22.31 ± 2.16	22 ± 1.92	0.778^NS^
	Co	53.64 ± 3.49	51.51 ± 2.22	0.199^NS^
	W	14.31 ± 1.37	13.6 ± 1.36	0.346^NS^
**Ti-6Al-4 V**	C	1.11 ± 0.84	0.34 ± 0.11	0.058^NS^
	O	0.69 ± 0.42	2.26 ± 1.22	0.141^NS^
	Al	5.34 ± 0.51	5.16 ± 0.45	0.484^NS^
	Ti	93.06 ± 2.34	92.07 ± 2.16	0.428^NS^
***P*** **-Value** ^*^		0.002^S**^	0.008^S^	

^*^ *P*-value comparison between two solutions (independent t-test).

^*^ *P*-value comparison of O between the two alloys in different solutions (independent t-test).

^**^ S= Statistically significant at *P* ≤ 0.05; NS = Non-significant *P* < 0.05.

### Surface morphology

**Fig. 4:**
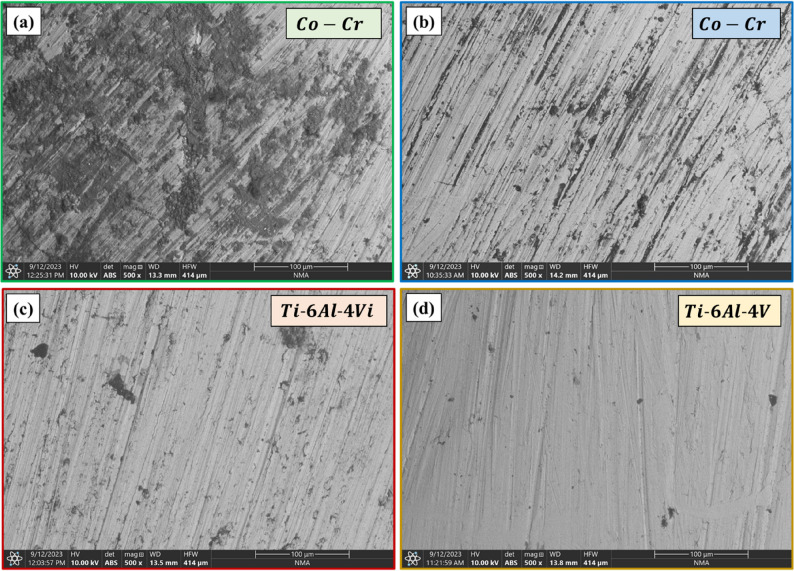
SEM micrographs of Co–Cr and Ti–6Al–4 V surfaces after immersion tests in (**a**, **c**) acidic and (**b**, **d**) neutral solutions

**Fig. 5:**
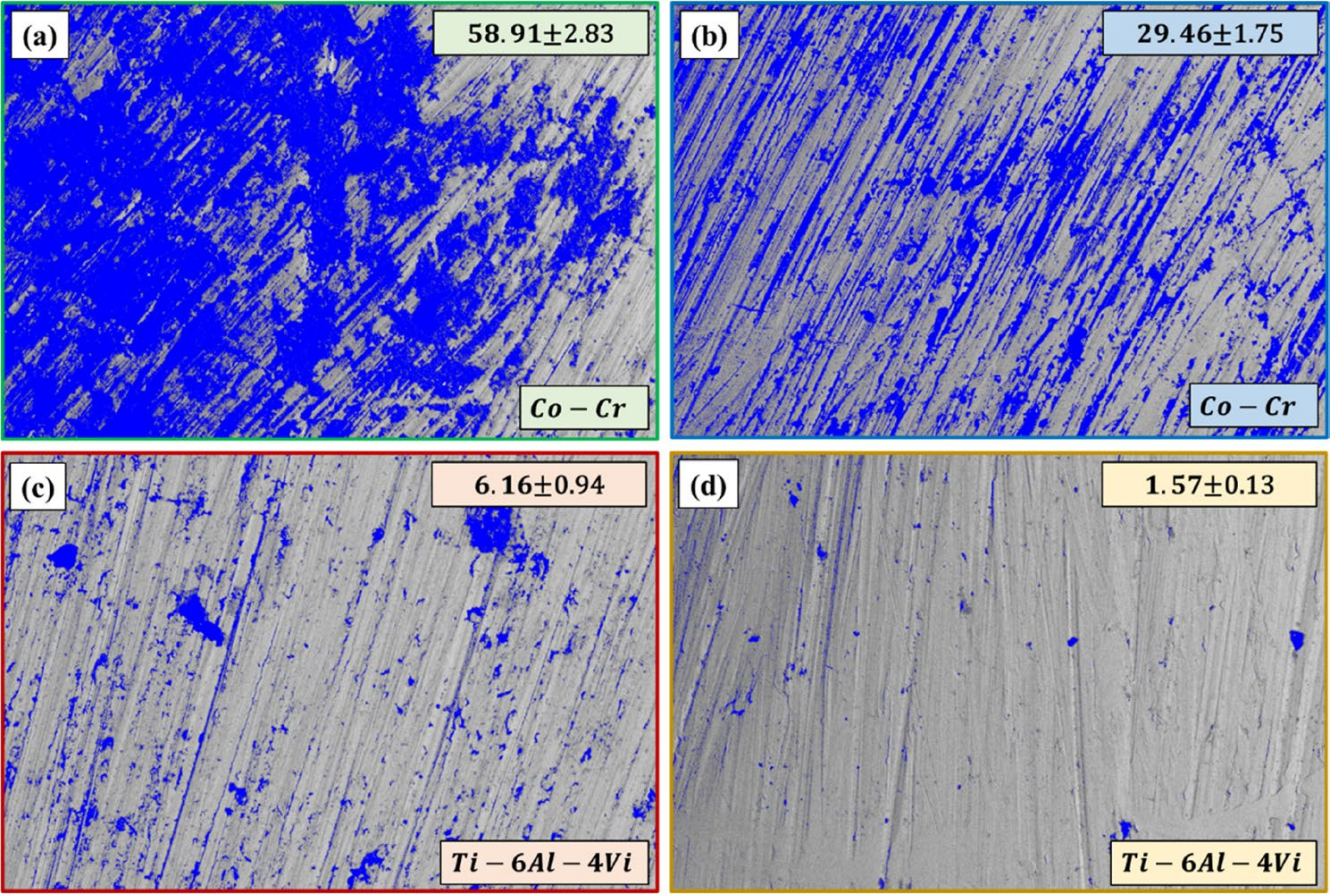
SEM micrographs of oxides deposited on the surfaces of Co-Cr and Ti-6Al-4 V after immersion tests in (**a**, **c**) acidic and (**b**, **d**) neutral solutions

**Fig. 6:**
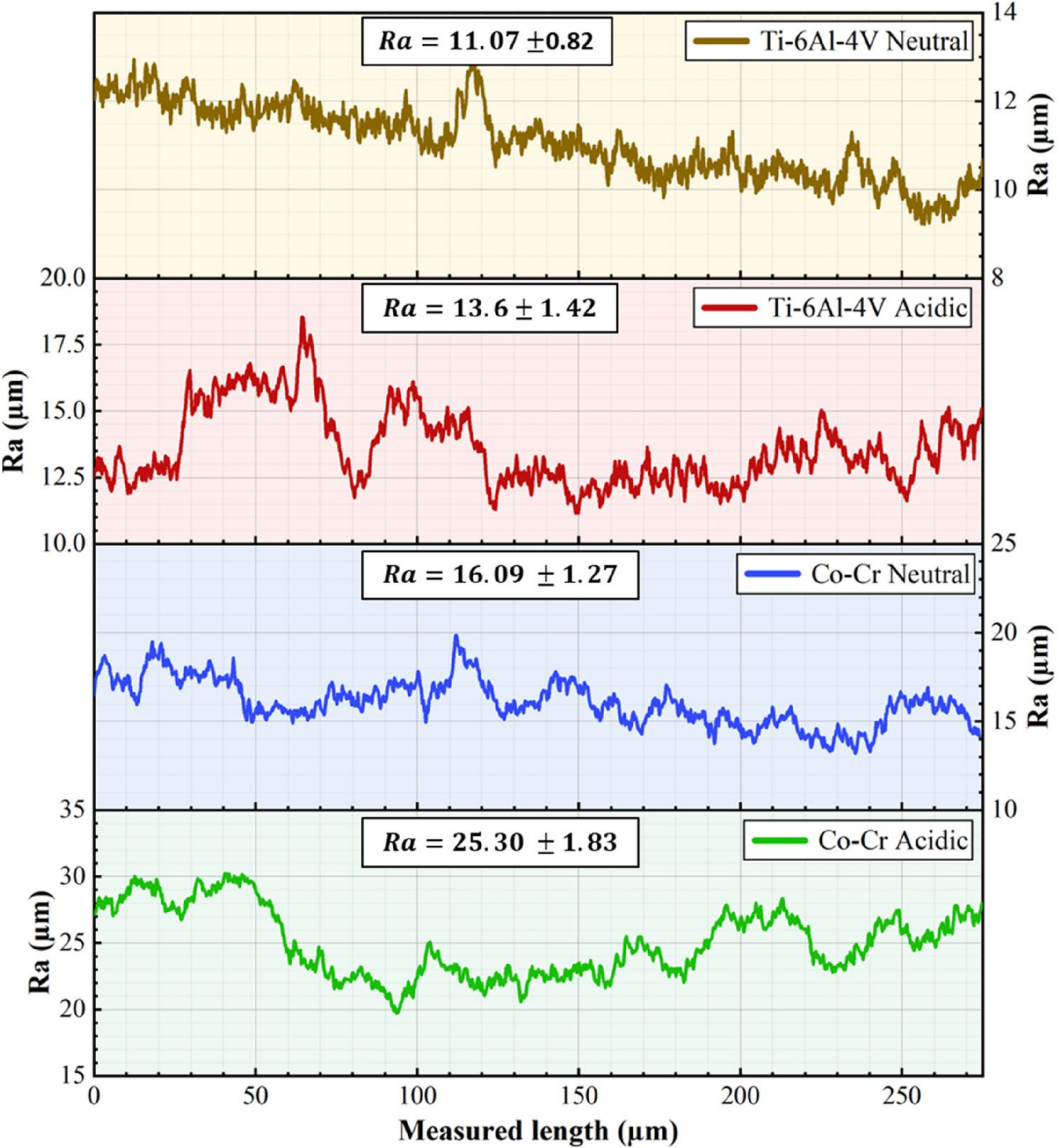
Surface roughness profile and Ra values of Co-Cr and Ti-6Al-4 V alloys after immersion tests in acidic and neutral solutions

SEM images of the tested alloys (show formation of oxide layers on the Co-Cr and Ti-6Al-4 V alloy surfaces when exposed to acidic and neutral solutions). It can be remarked that more extensive oxide (black area, Fig. 4, and blue area, Fig. 5) deposits formed on the Co-Cr surfaces than on Ti-6Al-4 V, especially in acidic solutions. Furthermore, both alloys demonstrated higher resistance to oxide formation in neutral solutions than in acidic solutions. Oxide formation appeared as distinct surface features that altered the topography of the alloy surfaces. For the Co-Cr alloy (Figs. 5a and b), there was a dramatic increase in oxide formation when exposed to the acidic solution compared to that in the neutral solution. The percentage of deposited oxides increased from 29.46% in neutral solution to 58.91% in acidic solution. On the other hand, the percentage of oxides increased in Ti-6Al-4 V alloy from 1.57% in neutral solutions to 6.16% in acidic solutions (Fig. 5c and d). These observations of oxide formation align with the EDS results in Table 3,

Figure 6; Table 4 show the surface roughness characteristics of the Co-Cr and Ti-6Al-4 V alloys exposed to acidic and neutral solutions, respectively. The data revealed significant differences in the responses of the two alloys to different pH solutions, particularly in terms of surface roughness. For the Co-Cr alloy, there was a substantial increase in surface roughness when exposed to the acidic solution compared to the neutral solution. The increased roughness suggests that the acidic solution likely caused more aggressive corrosion or etching of the Co-Cr surface. In contrast, the Ti-6Al-4 V alloy demonstrated much greater stability in terms of surface roughness under both pH conditions. Comparing the two alloys, it is evident that the Co-Cr alloy consistently exhibited a higher surface roughness than the Ti-6Al-4 V alloy under both neutral and acidic conditions. The difference in Ra between the two alloys was highly statistically significant (*p* < 0.001) in both pH solutions. This suggests that even under neutral conditions, the Co-Cr alloy has an inherently rougher surface than the Ti-6Al-4 V alloy, a characteristic that becomes even more pronounced under acidic conditions. These observations of oxide formation align with the EDX results in Table 2, which showed a significant increase in oxygen content for the Co-Cr alloy under acidic conditions, while the Ti-6Al-4 V exhibited a smaller, non-significant increase.

**Table 4:** Mean ± SD of Ra in different solutions pH (acidic and neutral solutions) for both alloys

Alloy	Acidic	Neutral	*P*-value*
Co-Cr	25.30 ± 1.83	16.09 ± 1.27	< 0.001^HS^
Ti-6Al-4 V	13.60 ± 1.42	11.07 ± 0.82	0.838^NS^
*P*-value**	< 0.001^HS^	< 0.001^HS^	

* *P*-value comparison between two solutions (independent t-test)

** *P*-value comparison of Ra between the two alloys in different solutions (independent t-test)

*HS* Highly significant at *P* ≤ 0.001

*NS * Non-significant *P* < 0.05

### Surface topography

**Fig. 7:**
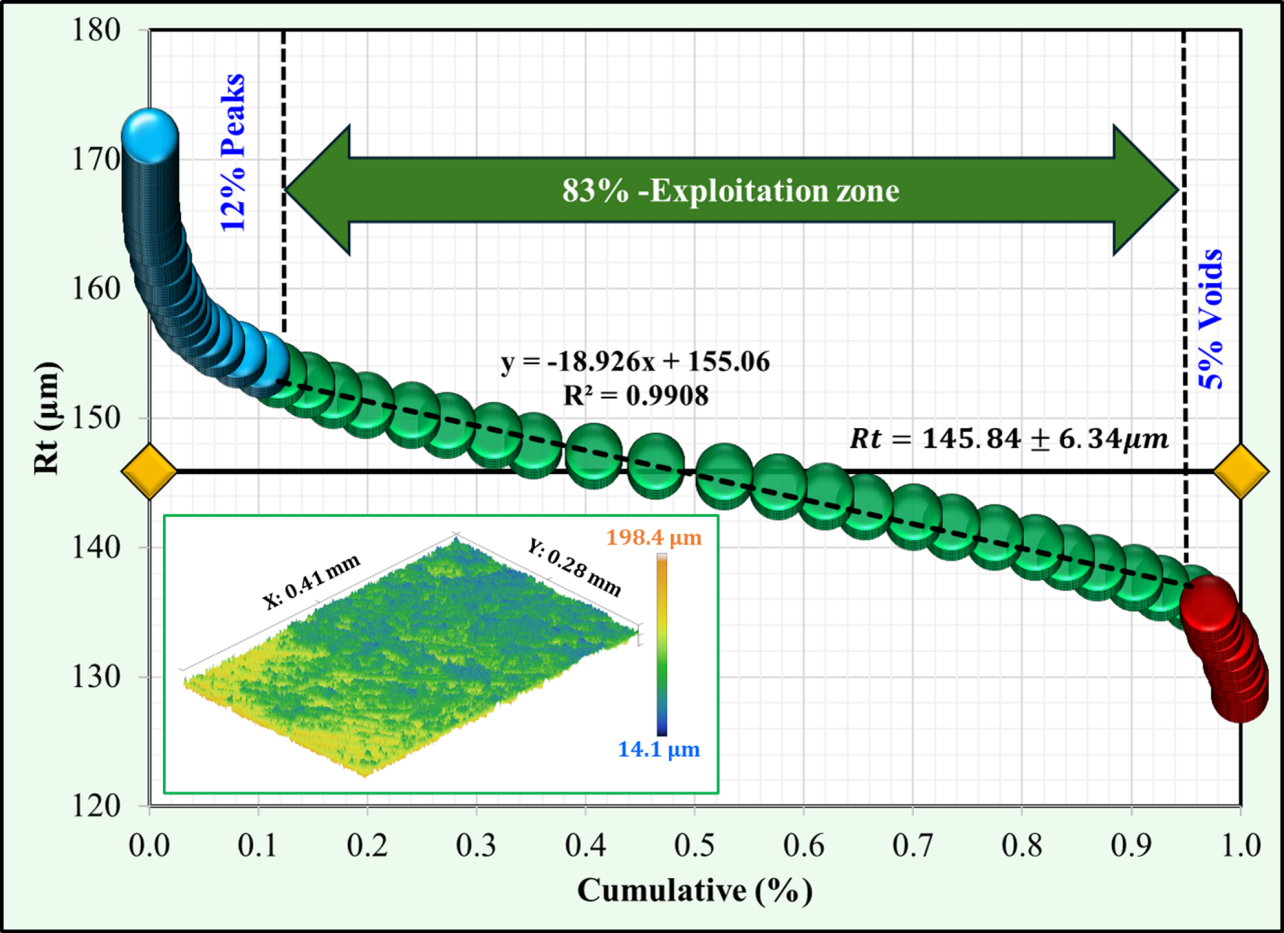
Surface topography of the Co-Cr surface after immersion in an acidic solution

**Fig. 8:**
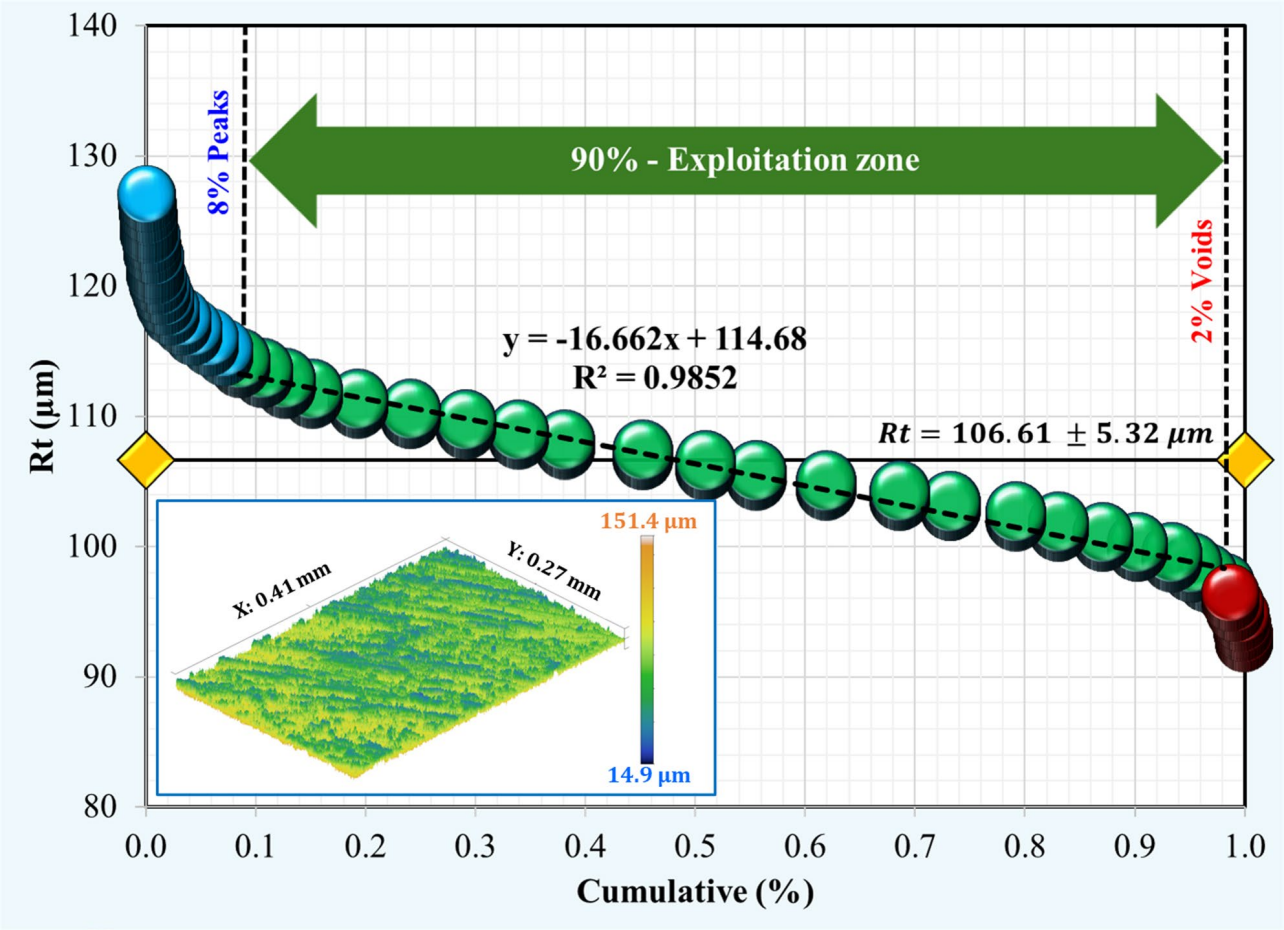
Surface topography of the Co-Cr surface after immersion in a neutral solution

**Fig. 9:**
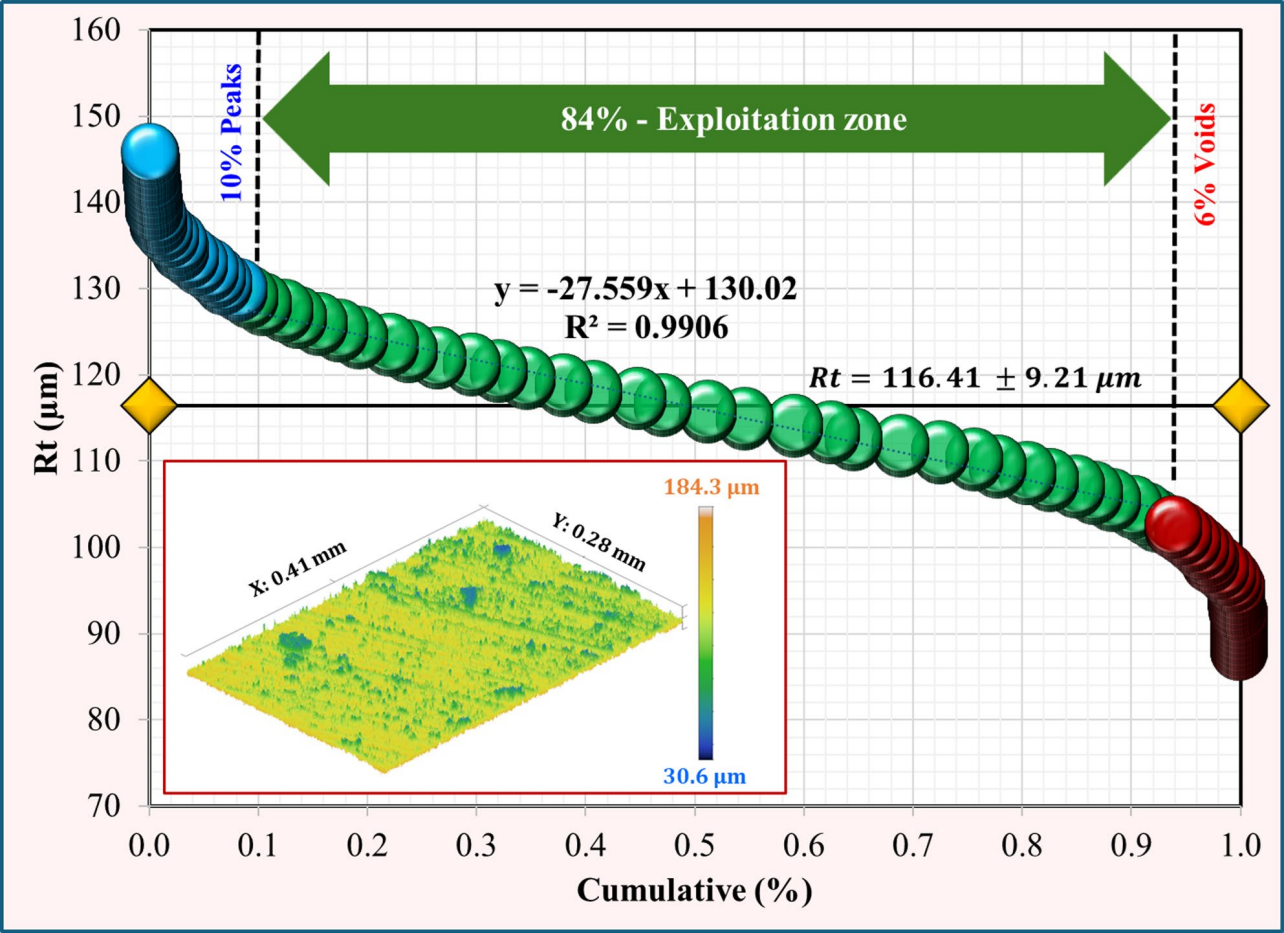
Surface topography of Ti-6Al-4 V after immersion in an acidic solution

**Fig. 10:**
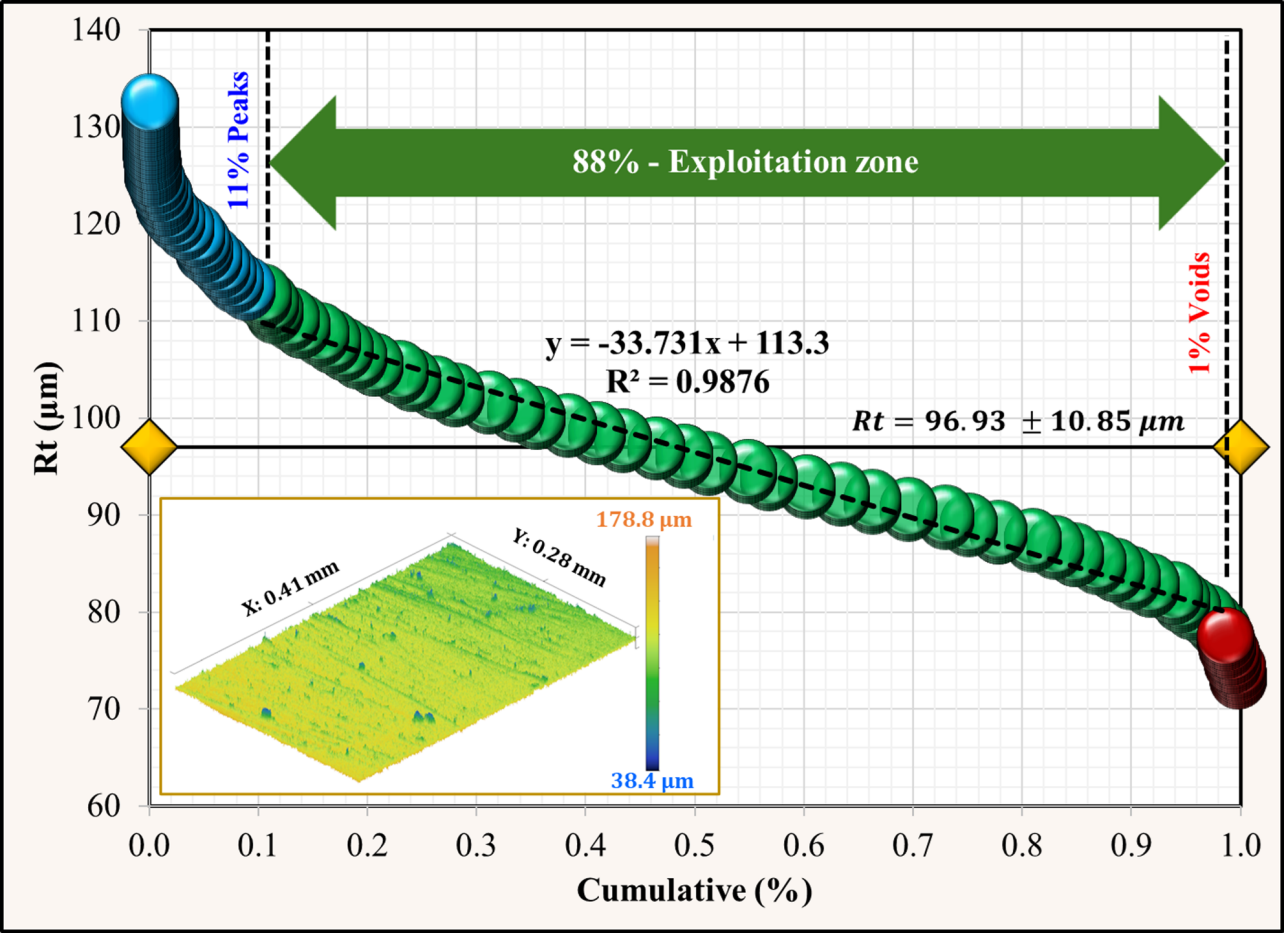
Surface topography of Ti-6Al-4 V after immersion in a neutral solution

Using the Abbott–Firestone approach, surface topography analysis was applied for the Co-Cr and Ti-6Al-4 V surfaces after immersion tests in acidic and neutral solutions. For the Co-Cr alloy (Figs. 7 and 8), the percentage of peaks decreased from 12% in the neutral solution to 8% in the acidic solution, indicating a rougher surface. Additionally, the void percentage was less than half, ranging from 5% to 2%. The exploitation zone, which represents the primary load-bearing area, increased from 83% to 90%. These changes collectively resulted in a higher total roughness (Rt) of 145.84 μm in the acidic solution compared to 106.61 μm in the neutral solution. Ti-6Al-4 V alloys exhibited different behaviors under neutral conditions, as shown in Figs. 9 and 10. Unlike Co-Cr, Ti-6Al-4 V showed a slight increase in the peak percentage from 10% to 11% under neutral conditions. However, the void percentage decreased significantly from 6% to 1%. The exploitation zone increased marginally from 84% to 88% of the total area. The total roughness (Rt) decreased from 116.41 μm under acidic conditions to 96.3 μm under neutral conditions. For the Co-Cr alloy, the percentage of peaks decreased significantly from 12% in acidic solution to 8% in neutral solution. This represents a 33% decrease in peak formation when exposed to an acidic solution. For the Ti-6Al-4 V alloy, the percentage of peaks increased slightly from 10% in the acidic solution to 11% in the neutral solution. The substantial reduction in the peak percentage suggests that the Co-Cr alloy experiences considerable surface roughening under acidic conditions, potentially leading to a more irregular surface topography. The minor 1% change in peak formation in the Ti-6Al-4 V alloy between acidic and neutral solutions indicates that the Ti-6Al-4 V alloy maintains a more stable surface topography even when exposed to acidic conditions, showing a lower tendency for surface roughening. Comparing the two alloys, it is evident that the Co-Cr alloy experienced a more dramatic change in the peak formation when exposed to acidic solutions. The 50% increase in Co-Cr compared to the 9% decrease in Ti-6Al-4 V suggests that Co-Cr may be more susceptible to surface alterations under acidic conditions. This could potentially lead to increased surface roughness and irregularities over time. These results were confirmed by morphological analysis “Surface Morphology” section. For the Co-Cr alloy, the exploitation zone increased significantly from 83% in the acidic solution to 90% in the neutral solution. This represents a 7% improvement in the primary load bearing area under acidic conditions. However, the Ti-6Al-4 V alloy demonstrated greater stability in the exploitation zone. The exploitation zone increased marginally, from 84% in the acidic solution to 88% in the neutral solution, representing an improvement of only 4%. This smaller change, compared to that of the Co-Cr alloy, indicates that the Ti-6Al-4 V alloy maintained a more consistent surface topography and load-bearing area, even when exposed to acidic conditions. In addition, both alloys maintained a higher exploitation zone of over 88% in a neutral solution, suggesting potentially better wear resistance, load-bearing capacity during chewing food processing, and a long lifetime under neutral conditions. For the Co-Cr alloy, the void percentage decreased from 5% under neutral conditions to 2% under acidic conditions. This represents a 60% decrease in the void percentage when exposed to a neutral solution. In contrast, the Ti-6Al-4 V alloy exhibited a greater reduction in the void percentage. Under acidic conditions, the void percentage was 6%, which decreased to 1% when exposed to a neutral solution. This represents an 83% decrease in the void percentage, which is significantly higher than the change observed in the Co-Cr alloy.

## Discussion

The present study investigated the effect of simulated gastric acid on the electrochemical behavior, surface morphology, and surface topography of additively manufactured Co-Cr and Ti-6Al-4 V dental alloys. Treatment protocols were designed to simulate the acidic conditions experienced in the oral cavities of patients with gastroesophageal reflux disease (GERD). The experimental methodology was implemented based on previous studies [1, 17] with modifications to mimic the oral environment. However, the simulated intermittent gastric acid exposure protocol was intentionally designed as a worst-case scenario to maximize corrosive challenge. On the other hand, distilled water was selected to provide a neutral and controlled environment, thereby isolating the effects of acid exposure without introducing additional ions or organic components present in artificial saliva. This approach allowed changes to be attributed primarily to acidic treatment.

The enhanced corrosion resistance of Ti-6Al-4 V shown in this study is in accordance with the well-known electrochemical stability of Ti-6Al-4 V alloys in physiological environments. Ti-6Al-4 V alloys naturally form a dense and adherent TiO₂ passive film that acts as an effective protection against electrochemical dissolution. This passive layer can self-repair when mechanically or chemically attacked, thereby maintaining long-term corrosion resistance even in harsh environments, including acidic and neutral solutions [18–21].Electrochemical studies have always shown that the stability of this oxide layer is the primary factor responsible for the excellent corrosion performance of Ti-based biomaterials [22, 23] .The presence of Al and V in the alloy further enhanced the stability and protective nature of the oxide layer, contributing to its improved corrosion resistance.

Furthermore, the electrochemical nobility of Ti-6Al-4 V is higher than that of Co and Cr, which inherently makes Ti-6Al-4 V less susceptible to corrosion in different pH environments [24, 25].This property, combined with the strong passive oxide layer, results in a lower corrosion current density (i_corr_) and corrosion rate (CR) for Ti-6Al-4 V compared to Co-Cr alloys. Although Co-Cr alloys also form a protective oxide layer, primarily composed of Cr2O3, it may not be as stable or protective as the oxide layer formed on Ti-6Al-4 V, especially in more aggressive acidic environments [26]. This difference in the oxide layer stability and protective properties explains the observed lower i_corr_ and CR values for Ti-6Al-4 V in both acidic and neutral solutions. The electrochemical results reported in this work correspond with previous studies comparing Ti and Co-Cr alloys in corrosion environments. For example, Şahin et al. [13] examined the corrosion behavior and ion release properties of Ti-6Al-4 V and Co–Cr dental alloys fabricated using CAD-CAM milling and laser sintering techniques. They reported that Ti-6Al-4 V alloys exhibited lower corrosion rates and greater electrochemical stability compared with Co–Cr alloys in simulated oral environments. Similarly, electrochemical impedance spectroscopy studies have demonstrated that Ti alloys generally exhibit higher charge-transfer resistance than Co-Cr alloys due to the stability of their oxide films. Manaka et al. [25] investigated galvanic corrosion behavior among Ti-6Al-4 V, Co-Cr-Mo alloys, and other biomedical metals. Their study indicated that Ti-6Al-4 V alloys possessed higher electrochemical properties and lower corrosion sensitivity compared with other alloys in simulated physiological solutions. These observations reinforce the interpretation that the electrochemical characteristics of Ti-6Al-4 V contribute to its improved corrosion resistance. The significant increase in oxygen content for Co-Cr indicates more extensive oxide formation, which is visually confirmed by SEM analysis showing larger oxide deposits on Co-Cr surfaces, particularly in acidic solutions. Oxide overgrowth in Co-Cr alloys typically produces a porous and non-adherent layer. Chloride ions penetrate small defects in the oxide film. This forms a localized electrochemical cell, leading to pitting corrosion, which increases surface roughness. This role of chloride is even amplified by low Ph [25]. Furthermore, Oxide over growth is often characterized by the incorporation of metastable cobalt oxides, which possess inferior chemical stability [27]. An increased thickness of the oxide layer may be associated with a higher degree of metal ion release. Although extended exposure to metal ions in the oral mucosa can promote systemic absorption and cause negative reactions, such as vanadium-induced cytotoxicity and cytokine release, Ti-6Al-4 V remains the clinical standard for biocompatibility; its vanadium leaching typically stays below toxic thresholds [28]. Likewise, increased ion release of Co–Cr alloy in acidic conditions doesn’t exceed the limits of cell viability according to standards [29]. Surface morphology analysis also supported the electrochemical findings of the present study. The higher level of oxide observed on Co–Cr surfaces after acidic exposure suggests that corrosion processes were more severe in this alloy due to the localized corrosion. This localized corrosion mechanism is especially relevant to Co-Cr alloys, whose Cr₂O₃ passive layer is susceptible to breakdown under the combined influence of low bulk pH and the further acidification occurring within pore microenvironments, potentially explaining the more extensive and heterogeneous oxide deposit distribution observed on Co-Cr surfaces. Similar observations have been reported in studies examining the corrosion behavior of dental alloys in acidic environments. Kassapidou et al. [30] evaluated Co–Cr alloys produced using different manufacturing techniques and reported increased surface roughness and higher ion release when exposed to corrosive media. They attributed these changes to localized corrosion processes and instability of the passive Cr oxide layer under certain conditions. The relatively stable surface morphology observed for Ti-6Al-4 V may be attributed to the strong protective effect of its passive oxide film. The higher resistance of Ti-6Al-4 V to localized corrosion can be attributed not only to the inherent stability of its TiO₂ passive film but also to the ability of this film to rapidly regenerate within pore microenvironments, effectively passivating localized corrosion sites before significant material loss occurs. Previous investigations have demonstrated that the TiO₂ layer provides significant protection against chemical degradation in acidic and biological environments. Mirza Rosca et al. [29] used electrochemical impedance spectroscopy to characterize Ti alloys containing various alloying elements and found that the passive oxide layer significantly increased corrosion resistance by limiting ion diffusion and electrochemical reactions at the surface. Surface roughness measurements in the present study also indicated that Co-Cr alloys experienced more pronounced surface changes following acidic exposure. Surface roughness plays a critical role in the long-term clinical performance of dental materials because rougher surfaces may promote bacterial adhesion and plaque accumulation. Lorenzetti et al. [22] investigated bacterial adhesion on titanium surfaces with different surface modifications and reported that increased surface roughness can significantly influence microbial attachment. Therefore, the increased roughness observed in the Co-Cr alloy under acidic conditions may potentially affect oral biofilm formation and long-term prosthetic performance.

The Abbott–Firestone analysis results for the Ti-6Al-4 V and Co-Cr alloys provide a valuable understanding of their topographical changes under different pH conditions, which have significant implications for dental applications. The more dramatic changes observed in the Co-Cr peak formation and exploitation zone under acidic conditions compared to Ti-6Al-4 V can be attributed to the different corrosion behaviors of these alloys. Ti-6Al-4 V typically forms a more stable and protective TiO2 layer that is resistant to breakdown, even in acidic environments. This protective layer helps to maintain the surface topography of Ti-6Al-4 V more effectively than that of Co-Cr alloys. The reduction in the void percentage observed for both alloys in neutral solutions is a positive finding for their dental applications. This change in surface topography can lead to potential resistance to bacterial adhesion and accumulation of food debris. Furthermore, the reduction in surface voids can also contribute to improved corrosion resistance by minimizing the areas where corrosive agents can accumulate and initiate localized corrosion is particularly relevant in the dynamic oral environment, where pH fluctuations and various chemical challenges are common [26, 27, 31]. It can be concluded that the Abbott–Firestone analysis results suggest that both Ti-6Al-4 V and Co-Cr alloys may offer improved resistance to bacterial adhesion and food debris accumulation under neutral conditions, which is highly beneficial for dental applications in patients with GERD. Moreover, the stability of Ti-6Al-4 V in acidic environments further substantiates its suitability for patients with GERD.

This study has several limitations. First, the absence of conventionally manufactured Co–Cr and Ti-6Al-4 V alloys as control groups restricts direct comparison with additively manufactured Co–Cr and Ti-6Al-4 V alloys. Second, the lack of quantitative metal ion release analysis impairs the correlation between electrochemical and surface changes and potential biological risks. Third, oral exposure to gastric acid in GERD patients is typically intermittent, buffered by saliva, and rarely sustained at low pH values used in the study. The absence of comparison with milder acidic environments (pH 3–5) further limits translational relevance. Future studies should therefore incorporate appropriate control groups, ion release measures, and more physiologically representative exposure regimens to strengthen the clinical applicability of findings. Fourth, that samples were stored in distilled water at 37 °C following acid exposure rather than in artificial saliva. Distilled water was intentionally chosen to provide a neutral and controlled environment, isolating the effects of gastric acid without interference from ions or organic components. However, this approach does not fully replicate the oral environment, where saliva plays a buffering and protective role. Future studies should therefore incorporate artificial saliva to enhance physiological relevance and improve the translational applicability of the findings. Fifth, that corrosion testing was based on a single electrochemical measurement for each tested material. Repeated measurements were not carried out to enable statistical comparison of corrosion parameters. Sixth a static thermal environment (37 °C), which does not fully replicate the dynamic conditions of the oral cavity.

## Conclusion

Within the limitations of this study, It was concluded that for dental restorations in patients with gastroesophageal reflux disease, 3D printed Ti-6Al-4 V is recommended as the more suitable alloy, owing to its superior corrosion resistance and more stable passive oxide layer compared to Co-Cr in both acidic and neutral environments. Acidic exposure caused significant surface degradation and increased oxygen content in Co-Cr, while Ti-6Al-4 V maintained greater topographical stability with minimal changes in roughness. Ultimately, the superior stability of 3D-printed Ti-6Al-4 V offers a critical safeguard for the clinical success of prosthetic rehabilitations in the challenging intraoral environments of GERD patients. By minimizing acid-induced surface degradation, this alloy potentially reduces the risk of biological complications and structural failure, thereby enhancing the long-term predictability of the treatment and the overall quality of life for the patient. While Ti-6Al-4 V shows promising surface stability that could theoretically benefit patients with GERD, these findings are currently limited to physical and material data. Without direct biological testing, any claims regarding reduced bacterial adhesion or food debris remain speculative.

## Future studies

The findings and limitations of the present study have determined several key objectives for future research. Future studies will include thermal cycling protocols and simulated gastric acid exposure to better simulate the thermally dynamic oral environment of GERD patients. In addition, evaluate the mechanical properties, including hardness, flexural strength, and elastic modulus, of 3D-printed Co-Cr and Ti-6Al-4 V alloys following acidic exposure. Future work will also address tribo-corrosion behavior under simultaneous mechanical loading and acidic conditions, as well as the long-term stability of passive oxide layers over clinically relevant timescales using advanced characterization techniques such as X-ray photoelectron spectroscopy (XPS). Further clinical and microbiological research is required to confirm these potential benefits.

## Data Availability

The datasets used and/or analyzed during the current study are available from the corresponding author upon reasonable request.
